# Understanding organisational development, sustainability, and diffusion of innovations within hospitals participating in a multilevel quality collaborative

**DOI:** 10.1186/1748-5908-6-18

**Published:** 2011-03-09

**Authors:** Michel LA Dückers, Cordula Wagner, Leti Vos, Peter P Groenewegen

**Affiliations:** 1 NIVEL-Netherlands Institute for Health Services Research, Utrecht, the Netherlands; 2 Impact, Dutch Knowledge & Advice Centre for Post-disaster Psychosocial Care, Amsterdam, the Netherlands; 3 EMGO Institute for Health and Care Research, Free University Medical Centre, Amsterdam, the Netherlands; 4 Department of Medical Decision Making, Leiden University Medical Center, Leiden, the Netherlands; 5 Department of Sociology, Department of Human Geography, Utrecht University, Utrecht, the Netherlands

## Abstract

**Background:**

Between 2004 and 2008, 24 Dutch hospitals participated in a two-year multilevel quality collaborative (MQC) comprised of (a) a leadership programme for hospital executives, (b) six quality-improvement collaboratives (QICs) for healthcare professionals and other staff, and (c) an internal programme organisation to help senior management monitor and coordinate team progress. The MQC aimed to stimulate the development of quality-management systems and the spread of methods to improve patient safety and logistics. The objective of this study is to describe how the first group of eight MQC hospitals sustained and disseminated improvements made and the quality methods used.

**Methods:**

The approach followed by the hospitals was described using interview and questionnaire data gathered from eight programme coordinators.

**Results:**

MQC hospitals followed a systematic strategy of diffusion and sustainability. Hospital quality-management systems are further developed according to a model linking plan-do-study-act cycles at the unit and hospital level. The model involves quality norms based on realised successes, performance agreements with unit heads, organisational support, monitoring, and quarterly accountability reports.

**Conclusions:**

It is concluded from this study that the MQC contributed to organisational development and dissemination within participating hospitals. Organisational learning effects were demonstrated. System changes affect the context factors in the theory of organisational readiness: organisational culture, policies and procedures, past experience, organisational resources, and organisational structure. Programme coordinator responses indicate that these factors are utilised to manage spread and sustainability. Further research is needed to assess long-term effects.

## Background

On an international level, policy makers, healthcare providers, professionals, researchers, and consultants have at least one thing in common: They share a universal need for knowledge about the diffusion of best practices in the hope that it contributes to the optimisation of healthcare delivery. This same need provided the impetus for organisation-wide diffusion and quality-improvement programmes that have been designed and implemented in health settings throughout the world in recent years. Examples include the Patient Care Notebook, the 100,000 Lives Campaign, the Framework for Spread in the Veterans Health Administration, and the state-wide collaboratives described by Leape *et al. *[1-4] These were all experiments in which potentially promising working methods were the subject of a dissemination plan. The programme dealt with in this article, Better Faster pillar 3, adds an extra dimension by linking spread and sustainability explicitly to organisational development. It was seen as a solution to the lagging implementation of quality-management systems and the diffusion of best practices [5,6]. The programme was a multilevel quality collaborative (MQC) based on a variety of quality-improvement collaboratives (QICs) [7,8] and a leadership programme for executives. Between 2004 and 2008, three hospital groups joined the MQC for a two-year period. In the first year, multidisciplinary teams participated in the QICs and implemented improvement projects. In the second year, the projects were to be disseminated over new units and patient groups within the hospitals. Whilst implementing the projects, hospitals were expected to develop an infrastructure with indicators, accountability, and feedback loops, enabling them to control the quality of processes and outcomes by continuous learning [9].

### Study objective

A recent MQC evaluation study showed that policy measures launched in the Dutch hospital sector since 2000 to overcome the lagging development of quality-management systems have been accompanied by an increase in hospital size and the further development of quality-management systems [9]. A longitudinal analysis suggests that the development trend of the MQC hospitals is steeper than the development within the other hospitals. This means that the quality management in MQC hospitals became more systematic [10]; however, it is unclear to what extent this generic tendency to organisational learning is reflected in the strategy for spread and sustainability adopted by the hospitals during the programme. The objective of this study is to describe how MQC hospitals sustained and disseminated quality methods and the improvements made. As such, the mix of elements mentioned thus far provides an opportunity to study, what Øvretveit *et al*. referred to as, the value of QICs as 'intentional spread strategy' [11]. Moreover, from an organisational development perspective, the study may also add knowledge to the underexplored field of sustainability [12].

### The MQC and its setting

In this section, MQC components and the selection procedure for MQC hospitals are described. The following terminology is used: 'programme coordinator' refers to the senior management staff members who play a crucial role in the progress and coordination of the entire programme in each hospital; 'external change agent' refers to the facilitators as well as to the designers of the MQC and its components at the unit and hospital level (see Table 1).

**Table 1:** Stimulating organisational development, sustainability, and dissemination of healthcare innovations in hospitals: interventions and their specific components at different levels

Intervention	Level	Specific components
Implementation of the multilevel quality collaborative (MQC)	Unit/team (Micro)	- Six collaboratives: Breakthrough projects implemented by multidisciplinary teams (features: team training meetings, knowledge about best practices, plan-do-study-act cycles, performance monitoring), supported and facilitated by external change agents
	
	Hospital (Meso)	- Leadership programme (strategic and tactical management), facilitated by external change agents
		- Internal hospital programme structure (supporting congruence between levels and to track progress)

Arranging supportive conditions/incentives in the environment of hospitals	National (Macro)^a^	- National focus/agenda setting (Better Faster topics)
		- Increased competition between hospitals (regulated market)
		- New reimbursement system for hospital care
		- Transparency (national set of performance indicators)

^a^Although the focus of this study is restricted to the MQC (micro and meso level), interventions at the macro level are relevant. The MQC was embedded in a broader policy mix and implemented in a sector where incentives and other measures were brought in gradually to induce hospitals to deliver high-quality, safe, patient-oriented, and efficient care [9,28-30].

#### Micro level: teams joining quality-improvement collaboratives

At the unit level, the MQC consists of different QICs. A QIC 'brings together groups of practitioners from different healthcare organizations to work in a structured way to improve one aspect of the quality of their service. It involves them in a series of meetings to learn about best practices in the area chosen, about quality methods and change ideas, and to share experiences of making changes in their own local setting' [11]. Within the MQC, teams were trained to apply 'Breakthrough' methods, requiring the application of plan-do-study-act (PDSA) improvement cycles and the answering of three questions: (1) What are we trying to accomplish? (2) How will we know that a change is an improvement? and (3) What change can we make that will result in an improvement? [13,14] QIC teams received organisational support and training from external change agents. They worked under time pressure and had to test several interventions while measuring their outcomes [11,14]. Table 2 shows the targets of the Breakthrough QICs. In each hospital, two series of roughly 10 projects had to be implemented. Every hospital had to assemble one or more teams per topic. Teams sometimes selected topics at their own initiative, while in other instances the areas were selected for them.

**Table 2:** Six collaboratives: targets and planned number of projects per hospital over two years

Breakthrough collaborative	Targets	Number of planned projects per hospital	
*Patient logistics*		**Year 1**	**Year 2**
Working without waiting lists (WWW)	Access time for outpatient appointment is less than a week	2	2
Operating theatre (OT)	Increasing operating theatre productivity by 30%	1	1
Process redesign (PRD)	Decreasing the total duration of diagnostics and treatment by 40% to 90% and length of in-hospital stay by 30%	2	2
*Patient safety*			
Medication safety (MS)	Decreasing the number of medication errors by 50%	2	2
Pressure ulcers (PU)	Percentage of pressure ulcers is lower than 5%	2	2
Postoperative wound infections (POWI)	Decreasing postoperative wound infections by 50%	1	0
**Total**		**10**	**9**

Source: *National strategy Better Faster pillar 3 *[6]

The typical team in the first year had eight members: two physicians, two nurses, one manager, a quality manager, and one or two members with topic-related expertise. Usually, a delegation of four team members visited four QIC meetings [15]. The external change agents created and presented training material, different interventions (optional), and outcome indicators to monitor progress and to run PDSA cycles (required). During the year, the teams chose and experimented with several interventions:

1. Pressure-ulcers teams measured the prevalence of pressure sores more systematically and regularly changed the position of the patient; risk factors were also assessed.

2. Medication-safety teams reduced unnecessary intravenous antibiotics, blood transfusions, and postoperative pain by applying guidelines on antibiotic usage, blood transfusions, and a visual linear pain-measurement instrument.

3. Operating theatre productivity focused on starting on time, clarified the definition of emergency, and reallocated extra operating time based on utilisation.

4. Postoperative wound-infection teams participated in an infection surveillance network, reduced the number of times that operating theatre doors were opened as well as the number of individuals present, and refined shaving procedures.

5. Waiting-list teams blocked agendas for six to eight weeks, anticipated fluctuations, and minimised consultations and consult types.

6. Process-redesign teams planned the diagnostic process in one or two days, balanced supply and demand, adopted interventions of the waiting-list project, and standardised process steps [16].

### Meso level: leadership programme and internal programme organisation

Instead of a single-level change approach, the literature suggests a strategy that involves actors at all organisational layers--from physicians and nurses to management and executives [17-20]. The MQC designers shared this perspective and included a leadership programme. The leadership and organisational development (L&O) strived to develop an improvement infrastructure at the hospital level, based on leadership and performance management [21]. The goal was to eventually align vision, quality norms, supportive measures, and processes and outcomes by making hospital units accountable for their results and by creating feedback loops between the layers [9]. Originally, the external change agents intended to test the competencies of CEOs, yet the CEOs refused and this element was removed from the leadership programme [15]. Each year, five or six L&O network meetings were organised, in which CEOs shared and discussed experiences related to change processes in their hospitals. Guest speakers presented models and information about the use and applicability of management instruments, such as business cases.

A second MQC component at the meso level was the installation of an internal programme structure with a central steering group and a programme coordinator. The internal programme structure was meant to keep organisational leaders informed about team progress via periodic project reviews, thus providing a practical link between hospital management and implementation processes.

### Hospital selection

All Dutch hospitals could apply for MQC membership, and the external change agents selected candidates for site visits. They spoke to CEOs, senior managers, and medical staff and checked eight criteria: (1) level of ambition, (2) experience with multidisciplinary projects, (3) committed strategic management, (4) actors at all levels in favour of participation, (5) willingness to appoint a programme coordinator, (6) sufficient resources and manpower at all levels, (7) implementation of a new reimbursement system on schedule (Diagnosis-related groups-based system with diagnostic treatment combinations), and (8) no significant contraindications [15].

This study focused on the first group of eight hospitals. Hospitals could apply for membership until 16 July 2004. The external change agency received 12 application forms. After the site visits, eight hospitals were selected.

## Methods

### Questionnaire and dissemination table (see additional file 1)

For the last 15 years, a validated measuring instrument has been used to measure the developmental stage of the quality-management systems of all hospitals in the Netherlands. A distinction is made in five focal areas (quality-policy documents, human resource management, protocols and guidelines, systematic quality improvement, and patient participation in quality-management activities) and four developmental stages: (1) orientation and awareness, (2) preparation, (3) experimentation and implementation, and (4) integration into normal business operations [10]. Because MQC and non-MQC hospitals could be identified, the measurements have proved to be helpful in determining developmental stages, assessing trends, and making group comparisons [9]. To gain insight in the relation between the strategy for spread and sustainability and quality-management system characteristics, in the second half of 2006, the programme coordinators from the first eight MQC hospitals received an additional questionnaire to measure

1. topics included in management contracts;

2. if units work with annual quality plans containing specific quality goals;

3. if units are expected to report results periodically, and if so, how often and to whom;

4. whether outcomes of different year-one projects are measured regularly.

By the end of 2006, the programme coordinators were asked to fill out a table with the second-year spread of QIC projects over new units and patient groups in their hospitals. The questionnaire items and the dissemination table are available in an additional file.

### Interviews

In autumn 2006, the programme coordinators were interviewed. Programme coordinators were likely to have the best knowledge of the overall implementation of the QIC projects and the support given by the organisation and the external change agency. They were consulted for information on system and process features that are considered to be relevant for systematic quality improvement and performance management [12,22]. The semistructured interview schedule contained five open questions:

1. How did your hospital organise the internal dissemination?

2. What kind of sustainability approach was followed?

3. What role did the internal programme coordinator and hospital executives play?

4. Were the targets on safety and logistics realised in all the project locations?

5. What is the most important lesson your hospital has learned from participation in the programme?

The focus of the interviews was on the hospital's supportive structure: vision, facilities to train new teams, the position of experts, how senior management kept track of project progress, and how up-to-date outcome information was generated in the units where projects were implemented. Programme coordinators were also asked about measures taken to spread and to sustain new ways of working and results.

Each interview was conducted by two researchers and lasted approximately one hour. Both interviewers made a report of the conversation. One of them made a first version of the interview report and the second interviewer checked this with his or her own transcription. After having reached agreement on the report, it was sent to the respondent who was requested to study the document and to assess whether it reflected the nature of the conversation and the topics dealt with. Based on the feedback, the interview report was corrected and finalised.

The content of the interview reports was coded. Codes were assigned, firstly, based on a study by Gustafson *et al*. emphasising the need for innovation to align with the organisation's overall strategy and mission, broad-based support and advocacy (from both top and middle management), attention to human resources (training and support), and meticulous monitoring of the impact of the change [23] and, secondly, on the categorisation of activities and components defining a quality-management system in its highest development stage: strategic quality action plans, training based on quality policy, systematic feedback, and management information systems [9,12].

From the perspective of organisational learning, the level of success of year-one projects was considered relevant to the second-year dissemination and received the code 'previous success'.

Specific safety and logistics targets explicitly displayed or formalised as norms, performance measurement, and reporting or feedback of results were coded, respectively, 'norms', 'measurement', and 'accountability'. 'Support' involves the supportive measures in place (*e.g*., training facilities, time, means, reward, attention, and advice) to enable the implementation of current and new project series. 'Information system' encompasses systems that provide timely, up-to-date, and accurate information. Cultural aspects--like the perceived relevance of shared values and beliefs of organisation members regarding the programme, the organisational strategy, or particular QIC projects--were coded 'culture'. 'Structure' includes those characteristics of the organisational structure that are essential to manage the programme.

The research protocol for this study has been reviewed by the Medical Ethics Review Committee of the VU University Medical Centre (registered with the US Office of Human Research Protections as IRB00002991). The committee gave approval for the study to be performed. The study does not fall within the scope of the Medical Research Involving Human Subjects Act.

## Results

### Questionnaire and dissemination table

Seven of the eight programme coordinators filled out the questionnaire. They all reported that production agreements and prevalence of pressure ulcers are part of the management contracts. Six of them mentioned patient satisfaction surveys and implementation of improvement projects. The access time for clinical consultation in days was mentioned by five programme coordinators, throughput times by three, and the prevalence of wound infections by two. According to the programme coordinators, most units in the first group of MQC hospitals had annual plans containing patient safety goals (mentioned seven times), efficiency goals (mentioned six times), patient satisfaction and clinical outcomes (mentioned four times), and service quality (mentioned twice). On average, all seven hospitals made it compulsory that their units inform the executives four times a year (range 3 to 12) about the level of norm compliance. The outcomes of all first-year QIC projects were measured regularly--except for operating theatre productivity, since these teams were not given a measuring instrument (see Table 3).

**Table 3:** programme coordinator questionnaire data

	1	2	3	4	5	6	7
*Topics included in management contracts*							
Production	+	+	+	+	+	+	+
Patient satisfaction survey	+	+	+	-	+	+	+
Implementation of improvement projects	+	-	+	+	+	+	+
Pressure ulcers	+	+	+	+	+	+	+
Access time for clinical consultation	+	+	-	+	-	+	+
Wound infections	-	-	-	-	+	-	+
Throughput times	-	-	+	-	+	-	+
							
*Most units work with annual plan containing specific goals*							
Service quality	-	-	+	-	+	-	-
Patient safety	+	+	+	+	+	+	+
Clinical outcomes	-	+	+	-	+	+	-
Efficiency	+	+	+	-	+	+	+
Patient satisfaction	-	-	+	+	+	-	+
Other topics	-	-	+	-	+	-	-
							
*Units report results to strategic management*							
Periodically	+	+	+	+	+	+	+
Annual frequency	12	3	3	4	2	2	4
							
*Outcomes of year-one project are measured regularly*							
Pressure ulcers	+	+	+	+	+	+	+
Medication safety	+	+	-	-	+	+	+
Operating theatre productivity^a^	-	-	-	-	-	-	-
Postoperative wound infections	+	+	+	+	+	+	+
Process redesign	+	+	+	+	+	+	+
Working without waiting lists	+	+	+	+	+	+	+

^a^An instrument to measure the main outcomes of operating theatre productivity was not available in the first year.

Table 4 shows that in year one, more than 100 QIC projects were implemented. The second-year dissemination wave consisted of 297 projects. Medication-safety projects were disseminated the most operating theatre projects the least. The average number of projects disseminated over new processes and patient groups in the second year was 6.2. In 10 cases, a project was disseminated throughout the whole organization during the programme: pressure ulcers four times, medication safety three times, postoperative wound infections one time, and process redesign twice.

**Table 4:** number of first- and second-year projects

		Year 1			Year 2		Hospital wide	
***Project***	Projects	Mean	Range	Projects	Mean	Range	Year 1	Year 2
Pressure ulcers	20	2.5	2-5	55	6.9	0-20	1	3
Medication safety	17	2.3	1-4	95	11.9	0-23		3^a^
Operating theatre	8	1.0	0-0	5	0.6	0-2		
Postoperative wound infections	10	1.3	1-2	30	3.8	1-13		1
Process redesign	26	3.3	1-5	55	6.9	2-16		2
Working without waiting lists	26	3.3	1-5	57	7.1	3-14		
**Total**	**107**	**2.3**	**1-5**	**297**	**6.2**	**0-23**	**1**	**9**

^a^Four medication safety projects were disseminated hospital-wide, but only three cases happened during the multilevel quality collaborative. Unnecessary intravenous antibiotics was spread in two hospitals; in one of them in the second year, in the other before the programme. Unnecessary blood transfusions was spread in the second year in two hospitals.

### Interviews

The questionnaire shows that several system and process characteristics for systematic quality management are in place. Next, the interviews provide the narrative data needed to conceptualise how the strategy for dissemination and sustainability depends on the elements distinguished in the literature. Each of the elements (coded as culture, norms, measurement, accountability, previous success, support, information system, and structure) was mentioned by the programme coordinators.

Beginning with 'previous success', the programme coordinators suggested that the PDSA cycles Breakthrough projects are based on were also applied at the hospital level, with the goal of disseminating the projects over new units and checking whether results were maintained. It starts with implementing projects within a few pilot units. As soon as management is positive about the merits of these projects (*i.e*., goals are realised or substantial improvement has occurred) results are likely to be made a norm for other units:

The first year was less success-driven. Promising projects were identified on beforehand and planned by the tactical management. After the first pilots we decided: 'this is the way we are all going to work'. (Programme coordinator, hospital 8)

A second example:

You need a group of enthusiastic people. Improvement topics were chosen after pilot-testing. If an approach proves to be valuable then we consider making it obligatory. I look for such topics. Take working without waiting lists. The first year was not easy. We started in one unit. When it turned out to be a success, it became part of the hospital policy. (Programme coordinator, hospital 3)

However, earlier successes do not determine everything:

For the second year we took the potential of units into account. This was a bit of a puzzle. Eventually you want to implement each project throughout the hospital, but in practice, the number of projects depends on the available amount of support and the situation within the units. Baseline measurements are useful in this respect. (Programme coordinator, hospital 7)

Interviewees considered 'culture' highly relevant:

It is essential to generate internal support. By communicating successes within the hospital, physicians will initiate a project. Informal contacts are important. (Project coordinator, hospital 3)

Despite the tendency towards a system-driven push approach, MQC hospitals recognise their limits:

In those cases where people or units were not flattered by this strategy, another tactic was followed in which initial goals were maintained. Some people in this hospital appreciate a strong coordinating power, others do not. We listen to them and give room to unit-specific preferences. (Programme coordinator, hospital 1)

Another coordinator added:

In practice, internal dissemination is a combination of own initiative and obligation, of 'bottom up' and 'top down', and also of 'what do you want' and 'what can we offer' when it comes to supporting projects. (Programme coordinator, hospital 7)

The system-driven push approach depends on the elements norms, accountability, measurement, and information system. Coordinators provided numerous examples:

Several contracts are used, illustrating what the management expects from hospital staff and how things are to be organized in detail. (...) The annual board letter is very important in this respect. Goals are formulated within the hospital and therefore relevant and legitimate. The board of executives and the chief of the medical staff provide this legitimacy. (Programme coordinator, hospital 8)

The hospital uses management contracts and progress is discussed regularly with the board of directors. (Programme coordinator, hospital 5)

Agreements are made on internal spread. Hospital units are bound by higher-level management agreements that are linked to performance contracts. This is how spread and sustainability are positioned in the organisational structure. (Programme coordinator, hospital 6)

All respondents were convinced that spread and sustainability depend on the structural measurement of performance indicators, made accessible through management information systems:

Measurements are crucial. Teams must report their results, and give an indication of the time and frequency of the follow-up measurements. They will have to keep measuring the outcome indicators. The unit manager is responsible for this and the hospital management checks and ensures that it happens. A management information system is being constructed, containing production parameters and quality parameters. These are displayed on a 'dashboard'. The final indicators are decided upon together with the responsible physician. (Programme coordinator, hospital 7)

Another example:

A monitoring system has been established. Pressure sores, waiting lists, pain and wound infections are measured on a weekly basis. The measurements are imported in the system. (Programme coordinator, hospital 5)

As such, the goal of institution-wide diffusion is incorporated in the strategic policy of each hospital, linked to performance monitoring:

Better Faster became part of the hospital policy. Every quarter of the year data are reported. Outcomes will eventually be visualised on some sort of 'dashboard'. There are indicators for each topic. (...) The dashboard is the most important sustainability instrument. (Programme coordinator, hospital 3)

In this respect, programme coordinators stressed the importance of ICT systems and the need to further develop them:

Many hospitals are not equipped for systematic data collection. The tempo in which outcome measures have become more important, is much higher than the possibilities for measurement, registration, generating informative overviews, and using them. This is not a unit level responsibility. The organisation must make sure that the required systems are available. (Programme coordinator, hospital 8)

The support element affects these systems, but support means more than that. Units should receive the resources necessary to ease the implementation:

New teams are trained by those who have gained experience with a similar project, but we also provide other types of support. At the start of a project we determine how much support or training is needed and who is able to provide it. We assume there is sufficient expertise available within the hospital to support projects. (Programme coordinator, hospital 6)

Other coordinators confirmed the relevance of organisational support and approaches to reutilise knowledge and experiences:

A solid support platform is necessary for dissemination. In the first year some staff members fulfilled the role of process coach. They made use of the national expertise and QIC training offer. In the second year, they served as internal experts. (Programme coordinator, hospital 8)

In the second year, the MQC training model, as ran by the external change agents, was adopted by hospitals. New teams followed internal training programmes:

It works fine. Teams are formed that attend training meetings. The sessions take half a day, and are shorter than the ones given during Better Faster. The content, however, is not to be compromised. Elements we copied exactly are: the Breakthrough topics and working methods, reporting of progress, and measurement formats and instructions. (Programme coordinator, hospital 7)

The training offer depends on what people want. It requires tailoring. (Programme coordinator, hospital 5)

One of the last elements is structure. Various structural aspects have been addressed by the cited programme coordinators. The interviews point out that the hospitals intend to maintain the internal programme structure established during the programme. No signals were given that the hospitals intend to discard the approach with the elements as mentioned; they are essential to the strategy for spread and sustainability. There were, however, respondents who emphasised that the strategy is probably less suitable for some of the QIC projects:

We gave all topics a formal place except for process redesign, because these projects are more difficult to realise. Process redesign is a multidisciplinary story with less direct effects for hospital staff, making each implementation trajectory more difficult. Operation theatre productivity was not included in the dissemination scheme either. We have one large team working in three geographically separated hospital locations. Staff is exchanged between these sites. (Programme coordinator, hospital 4)

## Discussion

**Figure 1:**
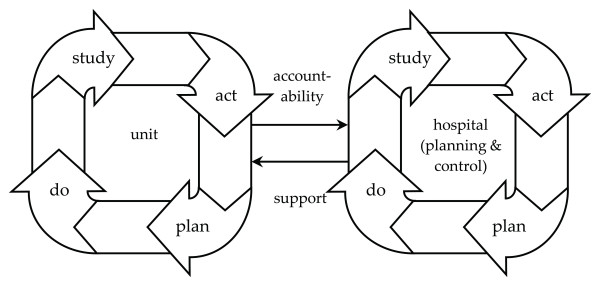
**model for organisation-wide dissemination: interactions between plan-do-study-act cycles at the unit and hospital levels**. The strategy for diffusion and sustainability begins with the implementation of projects in a few pilot units (left cycle). As soon as the hospital management (right cycle) notices that targets are realised or substantial improvement has occurred, these results are used as a baseline for other units. The new norm is added to the yearly policy documents that mark the start of the planning and control cycle. Within this framework, performance agreements are made with unit heads under the assumption that this will stimulate adoption of first-year lessons in an attempt to obtain similar results (again the left cycle). Both cycles are linked to each other. Units receive the means required for implementation (support), and on average, they report their progress to the management four times a year (accountability).

The current study adds insight into the mechanism by which MQC hospitals organised sustainability and internal dissemination. Within the MQC, the attention of executives and managers was linked to QIC projects at the unit level. The leadership of hospital executives did influence the extent to which the behaviour of QIC teams and physicians at the micro level was aimed at achieving MQC norms formulated at the macro level. The multilevel QIC encouraged executives to do this in two ways. Not only by--as pointed out in an earlier MQC evaluation [24]--stimulating physicians to join quality-improvement initiatives but also by adopting the organisational strategy for sustainability and dissemination as described in this article. According to programme coordinators, the further development of the quality-management system should be shaped following a model for organisation-wide diffusion and sustainability. The mechanism is visualised in Figure 1. Breakthrough projects are based on PDSA cycles, and the respondents suggested that these cycles also be applied at the hospital level, with the goal of disseminating the projects over new units and checking whether results are maintained. It starts with implementing projects within a few pilot units (left cycle). As soon as management (right cycle) is positive about the merits of these projects (*i.e*., goals are realised or substantial improvement has occurred) results are likely to be made a norm for other units. This is done formally by integrating the norm in the yearly policy documents that mark the start of the planning and control cycle and serve as a frame of reference for CEOs, management, and staff. Performance contracts are made with unit heads to stimulate the adoption of lessons learned from successful projects in an attempt to obtain similar results (again the left cycle). The support and accountability relations connect both PDSA cycles to each other. Units receive the means necessary to ease the implementation. On average, units report their proceedings to the management four times per annum.

This model requires that enough resources are made available and that accurate, timely data can be generated for the sake of accountability. That is to say, unit staff must be equipped to implement new working methods, and information systems should enable the organisation to track the unit's status and progress. Programme coordinators acknowledge that many efforts have been made to optimise hospital information systems. They are convinced that monitoring data are crucial to sustain results and keep the dissemination going.

### Organisational readiness

The elements culture, norms, measurement, accountability, previous success, support, information system, and structure are confirmed to fulfill a crucial rule within the strategy for spread and sustainability, as adopted by hospitals participating in the MQC. These elements are similar to the 'possible context factors' identified but left unaddressed in Weiner's theory of organisational readiness for change [25]. Weiner conceptualises how different factors influence each other and form a chain, starting with five possible context factors: organisational culture, policies and procedures, past experience, organisational resources, and organisational structure. These factors influence two precursors for organisational readiness. The first one is what Weiner calls 'change valence'. The more organisational members value the change as being needed, important, beneficial, or worthwhile, the more resolved they will feel to engage in the courses of action involved in change implementation. The second aspect is 'informational assessment'. When organisational members share a common, favourable assessment of task demands, resource availability, and situational factors, they share a sense of confidence that collectively they can implement a complex organisational change. Change valence and information assessment both contribute to 'organisational readiness', which is defined as a shared psychological state in which organisational members feel committed to implementing an organisational change and confident in their collective abilities to do so. Organisational readiness itself influences 'change-related efforts' (initiation, persistence, and cooperative behaviour) that, in turn, contribute to 'implementation effectiveness' [25].

Weiner presents his view on organisational readiness as a way of thinking, best suited for examining organisational changes where collective behaviour change is necessary in order to effectively implement the change and, in some instances, for the change to produce anticipated benefits. The successful internal dissemination and sustainability of QIC projects in units within MQC hospitals can be approached from this organisational readiness theory and its determinants earlier in the chain. The programme can be viewed as an instrument to utilise the configuration of context factors.

### Future research

The second-year dissemination wave consisted of almost 300 projects (see Table 3). Additional research is needed to determine the level of success of these projects, to answer the question of whether successful first-year QIC projects are disseminated more often than nonsuccesses, and to assess long-term effects. For now, the study illustrates that MQC hospitals acted in accordance with the intentions of external change agents. It also confirms that ongoing dissemination requires success stories [12,26]. This study adds insight in organisational development and dissemination processes within hospitals participating in an MQC. Extra measurements are needed to verify whether MQC hospitals continued their dissemination and sustainability strategy after the programme. Additional research is needed to replicate findings and to answer other relevant organisation-structure or culture-related questions within the context of the improvement and dissemination programmes that are designed and released in health sectors internationally. It is also essential to gain additional insights into the process and outcomes of QIC implementation or the practical use of QICs as a spread strategy. Besides dissemination, it remains important to perform studies on the merits of QICs compared to alternative improvement techniques, to explore the applicability of rapid-cycle improvement for different quality topics, and to ascertain if QIC teams perform better--in the short and the long run--within an organisation participating in a multilayered instead of a single-layered programme.

### Limitations

One limitation of this study is that it depends partly on information collected from programme coordinators who were active MQC participants; their answers are perhaps too positive. Moreover, the study design would have been stronger if information from external change agents who facilitated the QICs had been included. Another shortcoming is that, ideally, count data on second-year dissemination should be accompanied by information on the relative complexity of implementation efforts. Where medication safety and pressure ulcers stick to a 'simple' implementation of principles in new patient groups, process redesign requires tailoring and an extensive analysis of the consequences of changes for other units and processes within the hospital [27]. In this article, differences between QIC projects were not taken into account.

## Conclusion

It is concluded from this study that the MQC has contributed to organisational development and dissemination within participating hospitals. Organisational system changes within MQC hospitals are described in relation to implementation processes at the unit level. Organisational learning effects are demonstrated. As could be expected from hospitals with highly developed quality-management systems, the MQC hospitals followed a sustainability and spread strategy in which learning cycles were applied at the institution level to assess the discrepancy between unit performance and organisational quality norms copied from macro-level MQC targets (waiting lists, pressure ulcers, etc.). This form of organisational learning connects implementation processes at the micro level to management processes at the meso level, leading to new implementation processes at the micro level one year later.

## Competing interests

The authors declare that they have no competing interests.

## Authors' contributions

MLAD was responsible for designing the study; acquiring, analysing, and interpreting the data; and drafting the manuscript. As project leader of the independent programme evaluation, CW was responsible for the design of the study. LV acquired and analysed MQC data. CW, LV, and PPG assisted in interpreting the results and revising the manuscript for intellectual content. All authors have read and approved the final manuscript.

## Supplementary Material

Additional file 1 **Questionnaire and dissemination table**. A copy of the questionnaire and dissemination table used in the study. Click here for file
