# Supplementary material for: Understanding organisational development, sustainability, and diffusion of innovations within hospitals participating in a multilevel quality collaborative
Source: Implement Sci. 2011 Mar 9;6:18. doi: 10.1186/1748-5908-6-18 (PMC3065434; doi:10.1186/1748-5908-6-18)
Supplement: Additional file 1 — Questionnaire and dissemination table. A copy of the questionnaire and dissemination table used in the study. [file 1748-5908-6-18-S1.DOC]

**QUESTIONNAIRE**

1. Which topics are included in the management contracts of your hospital?

- production Yes/No
- patient satisfaction survey Yes/No
- implementation of improvement project Yes/No
- Better Faster topics Yes/No

2. Do most units work with annual plans containing the following quality goals?

- service quality Yes/No
- patient safety Yes/No
- clinical outcomes Yes/No
- efficiency Yes/No
- patient satisfaction Yes/No
- other topics Yes/No

3. Are units expected to report results periodically? If so, how often and to whom?

- returning accountability moments Yes/No
- annual frequency ....................................................
- to whom ....................................................

....................................................

4. Are outcomes of the following year-one projects measured regularly?*

- pressure ulcers Yes/No
- medication safety Yes/No
- postoperative wound infections Yes/No
- process redesign (throughput times) Yes/No
- working without waiting lists Yes/No

* A measuring format for operating theatre productivity was not available in the first year.

**DISSEMINATION TABLE**

Please register the number of units or patient groups where projects were implemented in the first and second year. Was the project implemented in all relevant locations in the hospital?

| ***Project*** | **Year 1**  **Number of units or patient groups** | **Year 1**  **Hospital wide** | **Year 2**  **Number of units or patient groups** | **Year 2**  **Hospital wide** |
| --- | --- | --- | --- | --- |
| Pressure ulcers | ............ | Yes/No | ............ | Yes/No |
| Medication safety | ............ | Yes/No | ............ | Yes/No |
| Operating theatre productivity | ............ | Yes/No | ............ | Yes/No |
| Postoperative wound infections | ............ | Yes/No | ............ | Yes/No |
| Process redesign | ............ | Yes/No | ............ | Yes/No |
| Working without waiting lists | ............ | Yes/No | ............ | Yes/No |
